# Revisiting the Cookie Theft Picture for Cognitive Impairment: Assessing Its Relevance for Discourse Analysis After Four Decades

**DOI:** 10.1002/alz70856_106522

**Published:** 2026-01-07

**Authors:** Sina Shafiyan, Kathleen Fraser, Frank Knoefel, Neil W. Thomas, Manuela Kunz, Bruce Wallace, Rafik Goubran, Anna Zumbansen

**Affiliations:** ^1^ University of Ottawa, Ottawa, ON, Canada; ^2^ Bruyere Health Research Institute, Ottawa, ON, Canada; ^3^ National Research Council Canada, Ottawa, ON, Canada; ^4^ Carleton University, Ottawa, ON, Canada; ^5^ AGE‐WELL NIH SAM3, Ottawa, ON, Canada

## Abstract

**Background:**

The Cookie Theft Picture Description Task (CTPDT) has long been used to assess cognitive impairments, measuring discourse elements such as Information Units (IUs) and Information Density (ID). IUs represent details about subjects, locations, and actions. ID is the ratio of IUs to total word count. Recent concerns have been raised about the CTPDT's relevance in modern clinical settings because of the outdated visual content of the picture.

This study compares discourse measures in participants with Mild Cognitive Impairment (MCI) and Healthy Controls (HCs) from two cohorts, 40 years apart (1980s vs. 2020s).

**Method:**

The 1980s cohort includes 17 MCI participants and 36 HCs from the DementiaBank corpus (Becker et al., 1994). The 2020s cohort has 11 MCI participants and 15 HCs. The number of IUs and ID scores were automatically calculated using Croisile et al. (1996) criteria, followed by a manual review. Differences between cohorts were assessed with t‐test and Mann‐Whitney U tests, depending on the data distribution. Discourse measures, including IUs, ID, Unique IUs, and Unique ID, were compared between the MCI and HCs groups in both cohorts. Unique IUs refer to how many of the 22 IUs were mentioned at least once, and Unique ID is the proportion of unique IUs relative to the total word count.

**Result:**

Significant differences were found in Unique IUs and Unique ID between cohorts. HCs in the 2020s cohort produced more Unique IUs than those in the 1980s cohort (t‐test, *p* = 0.010). MCI participants in the 2020s cohort had a lower Unique ID than those in the 1980s cohort (Mann‐Whitney, *p* = 0.022). No significant differences were observed for other discourse measures, including total IUs and ID, across cohorts.

**Conclusion:**

Our analysis found no significant differences in most discourse measures, including total IUs and ID. However, there were notable variations in Unique IUs for HCs and Unique ID for MCI. These findings suggest that while the CTPDT is still applicable today, caution is advised when using older databases to interpret results.

